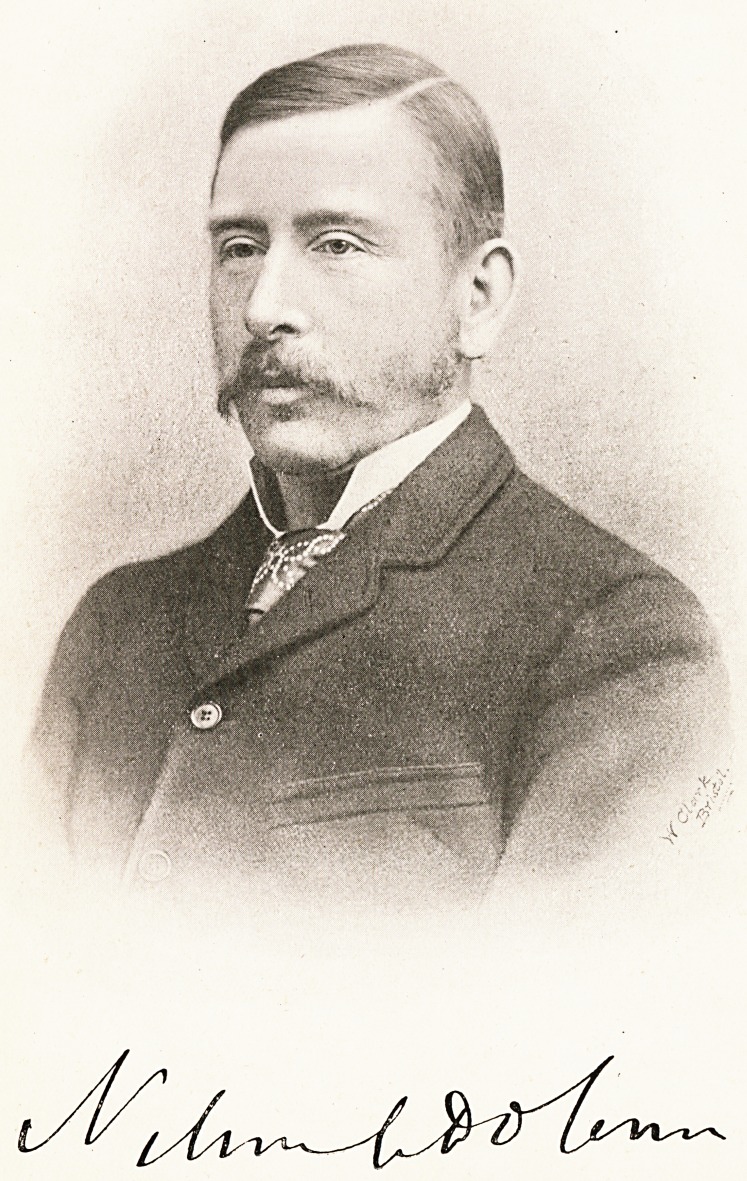# Nelson Congreve Dobson

**Published:** 1919-12

**Authors:** 


### Obituary.

### NELSON CONGREVE DOBSON, F.R.C.S., Ch.M. (Bris.). 1845–1919.

Mr. Dobson, whose life has been that of an invalid since the time when he resigned his office as Surgeon to the Bristol General Hospital in 1893, and afterwards dropped gradually out of active practice, was born at Holbeach, in Lincolnshire, in 1845.

His school life was passed in the Grammar School of that place, and at a private school in Cambridgeshire, but his progress was much retarded by continued ill-health. However, at 16 he was apprenticed (by the now extinct method) to a Dr. Harper of the town, and lived with him for three years. "He was an excellent surgeon for his time, and I learned many things of him for which I was always thankful." He then entered St. Thomas's Hospital Medical School, starting his student's career " with a fair knowledge of the bones and odds and ends of medical matters in general." Having gained many prizes as a successful student, he passed the M.R.C.S. and L.S.A. in 1867. He became House Surgeon at St. Thomas's, and was elected as Assistant House Surgeon of the Bristol General Hospital in 1867 and House Surgeon in 1868, but resigned the resident appointment after three years, when he commenced practice in Clifton in 1871, and was at once appointed Surgeon to the Bristol Royal Hospital for Sick Children and Women. This office he resigned within the year, on his election to that of Surgeon to the Bristol General Hospital, which he held with distinction until 1893, when he resigned in consequence of failing health, and became Consulting Surgeon to the Hospital.

One of his Hospital colleagues (Mr. C. A. Morton) writes as follows :—

" I can very vividly recall Mr. Dobson's activities as Surgeon to the General Hospital at the time when the most unfortunate affection of his sight began to interfere with his surgical work. He was so keenly interested in surgery, and in the welfare of his hospital patients, was so skilful as an operator, and so capable as a diagnostician, that his failing sight was a great calamity, not only to himself, but also to the Institution which he had so faithfully served for many years. We well remember the first indication of his imperfect vision. It was during the performance of an operation for varicose veins, when he asked his assistant to do more than is usually expected of an assistant, because, as he said, he could not see very clearly. But he always, in public at any rate, seemed to retain his cheerfulness of mind, though he knew what this failure of vision must lead to, and it was always a pleasure to be associated with him in his surgical work.

" Mr. Dobson was the first surgeon to suggest that a perforated gastric ulcer might be sewn up, though he had not the opportunity of putting his suggestion into practice. Sir Alfred Pearce Gould, in his address on this subject at the Bristol Annual Meeting of the British Medical Association, in 1894, soon after Mr. Dobson's retirement, referred to this, and also to the fact that Mr. Dobson's failing sight prevented his occupying the Presidential Chair of the Section of Surgery, which Sir Alfred then occupied.

" I well remember a discussion on Wheelhouse's operation for stricture, at a meeting of the Bristol Medico-Chirurgical Society at the time Mr. Dobson was in practice as a surgeon, and how he impressed us with the fact that this operation, which a surgeon had just very highly recommended, was not always, as he said, the ' very rosy operation ' which had been indicated, and he graphically described some of its difficulties and pitfalls.

"In 1889 Mr. Dobson gave the Presidential Address to the Bristol Medico-Chirurgical Society, in the formation of which he had been actively engaged, and with regard to its successful work he expressed the keenest satisfaction. In this address he made an eloquent appeal for early operation in suitable cases of cancer, but pointed out the inadvisability in many advanced cases. Like the surgeon of the present day, he says, 'I have constantly to deplore too late cases.'

"It is interesting to read in his address that he looked forward to the time when cancer should be as much under the control of medicine as syphilis; but, alas! though the treatment of syphilis by drugs has advanced considerably, yet there is no reason to think this is likely.

"The operations for cancer, when he was in practice, were by no means so extensive as to-day, and the surgeon did not even as a routine practice clear out the axilla when operating for cancer of the breast; yet Mr. Dobson tells us of a case in which he removed a cancerous breast without doing so, with the result that the patient was free from disease fifteen years later, although the malignant character of the growth seems to be without doubt, as it did recur soon after the operation in the scar, but the recurrent growth was removed. At this period of surgery (1889) Mr. Dobson and many other surgeons were deterred from operating in gastro-intestinal cancer by the very high mortality, and the rapid recurrence which seemed at that time to be frequent, and he did not consider operation advisable in such cases."

Meanwhile he had done useful service in the Bristol Medical School, having been Demonstrator and afterwards Lecturer on Anatomy and later on Surgery. Subsequently he became Professor of Surgery in the Bristol University College, and finally Emeritus Professor. Mr. Dobson passed the Fellowship Examination of the Royal College of Surgeons of England in 1870, and the honorary distinction Ch.M. of the University of Bristol was conferred on him in 1912. He did good work at the Hospital and Medical School, where as a fluent and ready speaker his presence was much appreciated by both his colleagues and the students who fell to his care. During his early years he took a considerable share in the negotiations and lengthened disputations which culminated in the foundation of the University College and the affiliation thereto of the old Bristol Medical School; this temporary affiliation scheme was followed by entire incorporation with the new college many years later, and afterwards with the newly-formed University of Bristol.

Mr. Dobson took quite a leading part in the establishment of the Bristol Medico-Chirurgical Society in 1874, he was a frequent contributor to the work of the meetings, and was duly appointed President for the year 1889–90. Many will remember his interesting Presidential Address on "Some Surgical Aspects of Carcinoma," where he concludes as follows: "It appears to me that in superficial cancer we have, up to the present, no remedy so reliable as early and free removal; and I end what I have to say by a plea for early and exact diagnosis and early reference to the surgeon."

He was also President of the Bath and Bristol Branch of the British Medical Association, and at the meeting of the Association in Bristol in 1894 was Vice-President of the Surgical Section.

For a long series of years Mr. Dobson took a warm interest in the Bristol Nurses' Institution and Private Nursing Home. He continued as Chairman of the Sub-Committee and Committee until within about a year of his death.

He was for many years a member of the Council of the University College of Bristol, and in 1895 he distributed the prizes and delivered an address to the students of the Medical Faculty.

Formerly he was a member and afterwards President of the Clifton Shakespeare Society, and this was a great comfort to him in his later invalided years, for he remembered much of what he had read, and was always ready with an apt quotation in the words of the great author.

In his active days he was fond of fishing, and enjoyed many a week-end testing the fishing resorts of the district; but the engagements of a large practice gave him little time for such amusements, and later the failure of his sight gave him little desire for it. The same remark applies to the pastimes of whist and bridge, the latter of which was a great solace to him for many years when he was not able to read, and whilst he still had some little vision with the help of a glass at short range.

Since the year 1898, over twenty years, his rôle has been that of a patient sufferer from maladies which he knew must be fatal in the long run; but his mental condition never failed till within a few days of his death, and he was always keenly interested in medical matters, as well as during the last few years in the daily events of the Great War. Having three sons in the Army and one in the Navy, the varying fortunes of the country were the subject of the keenest inquiry daily, and he felt most acutely the loss of two of his sons, one a brilliant officer who was killed in action at Loos, the other in Mesopotamia. The surviving son in the Army did much good work in India, Egypt, Arabia, and elsewhere. The naval son greatly distinguished himself recently in the attack on Kronstadt in the Baltic, and cheered the heart of the invalid at home by receiving the decoration of V.C., in addition to his previous D.S.O.

During the last year of his life, feeling his helplessness, he sometimes referred to his fate of blind inactivity.

> " We, like sentries, are condemned to stand
> In starless night, and wait the appointed hour."—DRYDEN.

When he contemplated—

> " That bourne from whence no traveller returns,"

a quotation from Spenser's *Faerie Queene* pleased him—

> " Our times are fix'd and all our days are number'd
> How long, how short, we know not. This we know,
> Duty requires we calmly wait the summons,
> Nor dare to stir till Heav'n shall give permission."

He often quoted from Hamlet's soliloquy—

> " To sleep, perchance to dream : ay, there 's the rub,
> For in that sleep of death what dreams may come
> When we have shuffled off this mortal coil."—Act iii., Scene 1.

Like Edwin Arnold, he appeared to await death with almost eager curiosity, as if anxious to find out the realities of the other side, for he would not accept the dreams of the spirit philosophers, whose so-called revelations he classed with Hamlet's " Words, words, words " (Act ii., Scene 1), and believed with Pope that—

> " Words are like leaves ; and where they most abound
> Much fruit of sense beneath is rarely found."

Dobson was not a prolific writer, but the following list would have been much extended if his sight had not failed him in comparatively early life.

BIBLIOGRAPHY.

" Ulcers Treated by Transplantation of Skin."—*Med. Times and Gaz.*, 1870, ii. 500.
" Removal of Greater Part of both Superior Maxillæ simultaneously for Malignant Disease."—*Brit. M. J.*, 1873, ii. 430.
" Two Cases of Surgical Interest."—*St. Thomas's Hosp. Rep.*, 1875, vi. 35.
(With W. A. Tilden.) " Therapeutic Value of the Crystalline Principles of Aloes."—*Med. Times and Gaz.*, 1876, ii. 177.
" Dentigerous Cyst in Lower Jaw."—*Tr. Bristol M.-Chir. Soc.*, 1878, i. 65.
" Lithotomy performed twice on same subject."—*Ibid.*, p. 73.
" Ovariotomy."—*Brit. M. J.*, 1879, i. 775.
" On Difficulties of Diagnosis in Abdominal Tumours."—*Obst. J. Gt. Brit.*, 1880, viii. 268.
" Tumours removed from proximity of the Subclavian and the Brachial Arteries."—*Brit. M. J.*, 1881, ii. 48.
" Sarcoma of Groin."—*Ibid.*, p. 856.
" Amputation in Senile Gangrene."—*Ibid.*, 1882, ii. 129.
" Abdominal Section in Perforating Ulcer of Stomach."—*Bristol M.-Chir. J.*, 1883, i. 196.
" Amputation at Shoulder Joint for Sarcoma of Biceps."—*Brit. M. J.*, 1884, i. 1091.
" Intestinal Obstruction."—*Bristol M.-Chir. J.*, 1885, iii. 28.
" Simultaneous Aneurysm of both Popliteal Arteries."—*Lancet*, 1885, i. 983.
" Large Aneurysm of the left Femoral Artery."—*Ibid.*, p. 984.
" Ten Years' Surgical Work."—*Med. Times and Gaz.*, 1885, ii. 566, 598.
" Suprapubic Lithotomy. Supra-vaginal Hysterectomy for Uterine Myoma."—*Lancet*, 1887, ii. 811.

"Cases of Oral Surgery."—*Bristol M.-Chir. J.*, 1889, vii. 108.
"Presidential Address on 'Some Surgical Aspects of Carcinoma.'"—*Ibid.*, p. 225.
"Multiple Tubercular Cerebral Tumours."—*Lancet*, 1892, i. 1079.
"Large Encysted Collection of Pus in Abdomen."—*Ibid.*, p. 837.
"Ruptured Papillomatous Ovarian Cyst."—*Ibid.*, p. 836.

## Figures and Tables

**Figure f1:**